# Exploring the Patient-Provider Relationship in Older Adult Pain Management

**DOI:** 10.1093/geroni/igab046.3391

**Published:** 2021-12-17

**Authors:** Sophia Sheikh, Jennifer Brailsford, Jason Beneciuk, Monika Patel, Brittany Johnson, Robin Moorman Li, Phyllis Hendry, Natalie Mitchell

**Affiliations:** 1 UF College of Medicine Jacksonville; 2 UF Health Jacksonville, Jacksonville, Florida, United States; 3 Brooks Rehabilitation and the College of Public Health & Health Professions at the University of Florida, Jacksonville, Florida, United States; 4 University of Florida College of Medicine – Jacksonville, Jacksonville, Florida, United States; 5 University of Florida College of Pharmacy, Jacksonville, Florida, United States; 6 UF College of Medicine Jacksonville, Jacksonville, Florida, United States

## Abstract

Successful health outcomes in older patients are linked to the quality of the patient-provider relationship. Our study objective was to further understand the role of this relationship specific to pain management through perspectives from older adults and healthcare providers. Semi-structured interviews and focus groups were conducted with 9 older adults and 11 multidisciplinary healthcare providers. Transcripts were analyzed using a thematic analysis. Three main concepts emerged: (1) defining pain management goals — differences in providers and patients’ goals for pain and function, with sub-themes of realistic goal setting and a shift in pain treatment to minimize opioids as a first-line medication; (2) communication — perceived gap in providers communicating and coordinating across disciplines and with patients, with sub-themes of improving positive communication and inconsistent messaging among providers; and (3) therapeutic alliance — all parties feel that developing a relationship is built on consistent trust and open dialogue. Although providers and older adults often expressed similar perspectives, there were several areas of misalignment identified within each concept, representing areas of disconnect within the patient-provider pain management relationship. Our findings indicate providers could benefit from education on improving communication around realistic goals and patient-centered outcomes and incorporation of more holistic pain management approaches when working with older adult patients. Further study should focus on developing educational interventions to address the identified shortcomings.

